# 3D multi-scale deep convolutional neural networks for pulmonary nodule detection

**DOI:** 10.1371/journal.pone.0244406

**Published:** 2021-01-07

**Authors:** Haixin Peng, Huacong Sun, Yanfei Guo

**Affiliations:** College of Computer Science and Engineering, Shandong University of Science and Technology, Qingdao, Shandong, China; Lingnan University, HONG KONG

## Abstract

With the rapid development of big data and artificial intelligence technology, computer-aided pulmonary nodule detection based on deep learning has achieved some successes. However, the sizes of pulmonary nodules vary greatly, and the pulmonary nodules have visual similarity with structures such as blood vessels and shadows around pulmonary nodules, which make the quick and accurate detection of pulmonary nodules in CT image still a challenging task. In this paper, we propose two kinds of 3D multi-scale deep convolution neural networks for nodule candidate detection and false positive reduction respectively. Among them, the nodule candidate detection network consists of two parts: 1) the backbone network part Res2SENet, which is used to extract multi-scale feature information of pulmonary nodules, it is composed of the multi-scale Res2Net modules of multiple available receptive fields at a granular level and the squeeze-and-excitation units; 2) the detection part, which uses a region proposal network structure to determine region candidates, and introduces context enhancement module and spatial attention module to improve detection performance. The false positive reduction network, also composed of the multi-scale Res2Net modules and the squeeze-and-excitation units, can further classify the nodule candidates generated by the nodule candidate detection network and screen out the ground truth positive nodules. Finally, the prediction probability generated by the nodule candidate detection network is weighted average with the prediction probability generated by the false positive reduction network to obtain the final results. The experimental results on the publicly available LUNA16 dataset showed that the proposed method has a superior ability to detect pulmonary nodules in CT images.

## Introduction

Lung cancer is one of the most dangerous malignancies to human health and life [1]. According to medical clinical experience, once the clinical symptoms of lung cancer show, the cure rate is very low, so the early detection of pulmonary nodules is of great significance for reducing lung cancer mortality [2]. As an important means of screening lung cancer in high-risk population, low-dose computed tomography scanning has been used in health examination on a large scale, but a large number of CT data have brought a lot of works to doctors and radiologists, and high-intensity works are easy to cause misdiagnosis of doctors. With the rapid development of big data and artificial intelligence technology, computer-aided detection based on deep learning has attracted wide attention [3].

At present, deep learning has achieved excellent results in the field of pulmonary nodule detection in chest CT sequence images. Zhu et al. [4] used Faster R-CNN [5] with dual path blocks and U-Net-like encoder-decoder structure for nodule candidate detection with the FROC (average sensitivity at the false positives as 0.125, 0.25, 0.5, 1, 2, 4, 8) score on the LUNA16 dataset [6] is 0.842. Dou et al. [7] used a 3D fully convolutional network [8] for lung nodule candidate detection and achieve a FROC score of 0.839 on the LUNA16 dataset, then use a residual network for false positive reduction. The sensitivity reaches 0.905 when the average number of false positives(FPs) per scan is 1. Khosravan et al. [9] proposed a pulmonary nodule detection network called S4ND, which consists of densely connected convolution blocks and is trained in an end-to-end manner, no post-processing is required to perfect the detection result and a FROC score of 0.897 can be achieved on the LUNA16 dataset. Xie et al. [10] adjusted the structure of 2D Faster R-CNN through two region proposal networks and an deconvolutional layer to detect nodule candidates with the highest sensitivity up to 0.864 on the LUNA16 dataset, then three 2D models are used to train three types of slices with different locations to reduce the number of false positive nodules with a FROC score of 0.790. Dou et al. [11] proposed a multilevel contextual 3D convolutional neural network for false positive reduction by using CT image cubes of different sizes as input and achieve a FROC score of 0.827 on the LUNA16 dataset. Wang et al. [12] concatenated three adjacent axial slices to construct 3D RGB images for nodule detection, the highest sensitivity can reach 0.968 when the average number of candidates per scan is 60.23 on the LUNA16 dataset, then false positives are reduced by two Inception-v4 networks [13] of different receptive fields with a FROC score of 0.903. Although the above methods have achieved good results, there is still room for improvement in sensitivity and false positives. How to use the data characteristics of CT sequence images to design more efficient network structure is the key to improve the performance of computer-aided detection systems.

This paper is based on deep convolutional neural network (DCNN). In order to improve system sensitivity and reduce false positives,we first use nodule candidate detection network to detect nodule candidates, and then use false positive reduction network to further classify nodule candidates to obtain final results. The main contributions of this paper are as follows:

1. CT image is composed of continuous sequence slices and 3D CNN can better capture the spatial information of CT sequence images and extract more abundant features. As a result, we designed two 3D deep convolutional neural networks, for detecting nodule candidates and reducing false positive nodules.
2. We created a 3D multi-scale pulmonary nodule detection network by embedding the squeeze-and-excitation unit [14] into multi-scale Res2Net [15] module of multiple receptive fields at the same granularity level, and introducing context enhancement module that integrates multi-scale features and spatial attention module that makes the network pay more attention to the regions of interest to improve the detection performance [16].
3. On the basis of multi-scale Res2Net modules and the squeeze-and-excitation units, a 3D false positive reduction network was created. We weighted average the prediction probability obtained by the false positive reduction network and the prediction probability obtained by the nodule candidate detection network to obtain the final result.
4. We validated the proposed method on publicly available LUNA16 dataset. Experimental results showed that our proposed method achieved competitive performance compared to several state-of-the-art networks. In addition, we conducted extensive ablation validation experiments to demonstrate the effectiveness of the method.

## Methods

**Fig 1:**
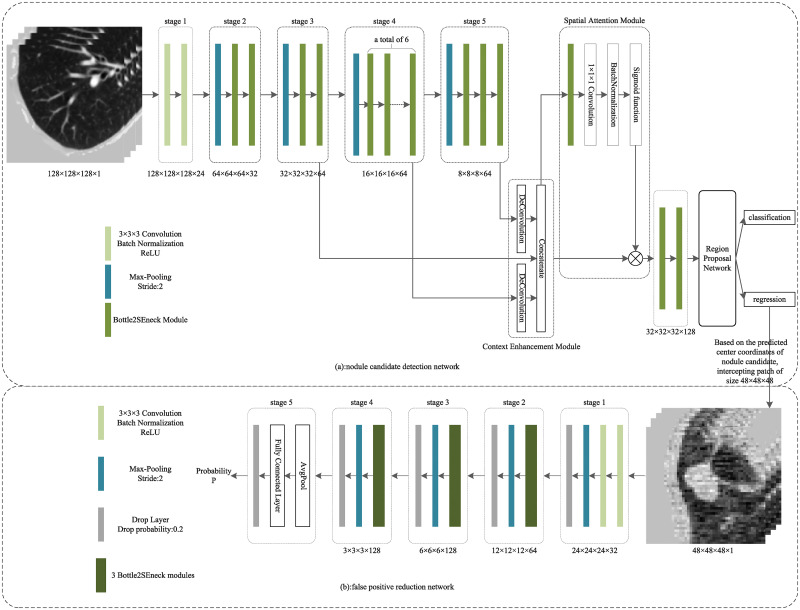
3D multi-scale pulmonary nodule detection networks. The CT images in Fig 1 were accessed through the links available on this page: https://luna16.grand-challenge.org/Download/ and had not been previously published and/or copyrighted.

The automatic detection of pulmonary nodules can be seen as an object detection task with input as CT image *I*, output as pulmonary nodule location information [*x*, *y*, *z*, *d*], where [*x*, *y*, *z*] represents the central coordinate of pulmonary nodule cube bounding box, *d* represents the diameter of pulmonary nodule. Our purpose in this task is to construct a mapping *F* from *I* to [*x*, *y*, *z*, *d*]. To achieve this goal, we proposed a 3D multi-scale pulmonary nodule detection network, as shown in Fig 1. The network consists of Bottle2SEneck modules and includes two parts: nodule candidate detection network and false positive reduction network.

### Bottle2SEneck

**Fig 2:**
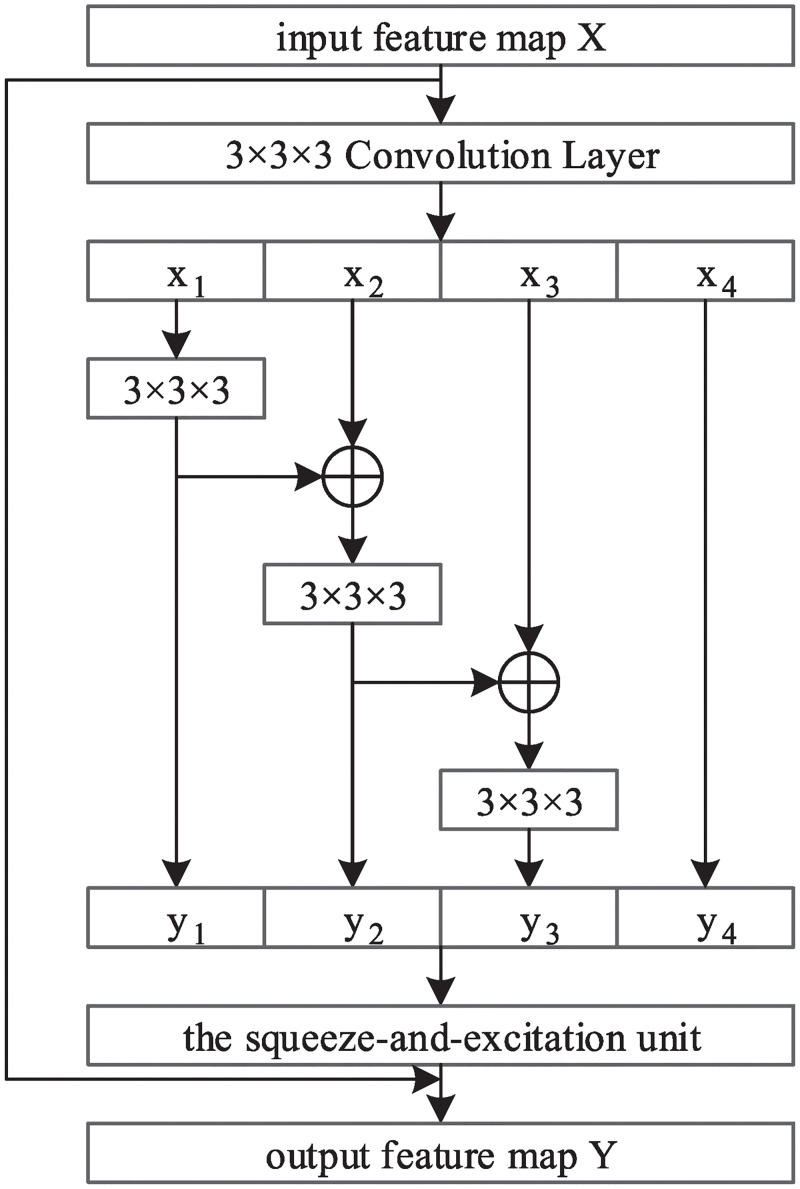
Bottle2SEneck module.

**Fig 3:**
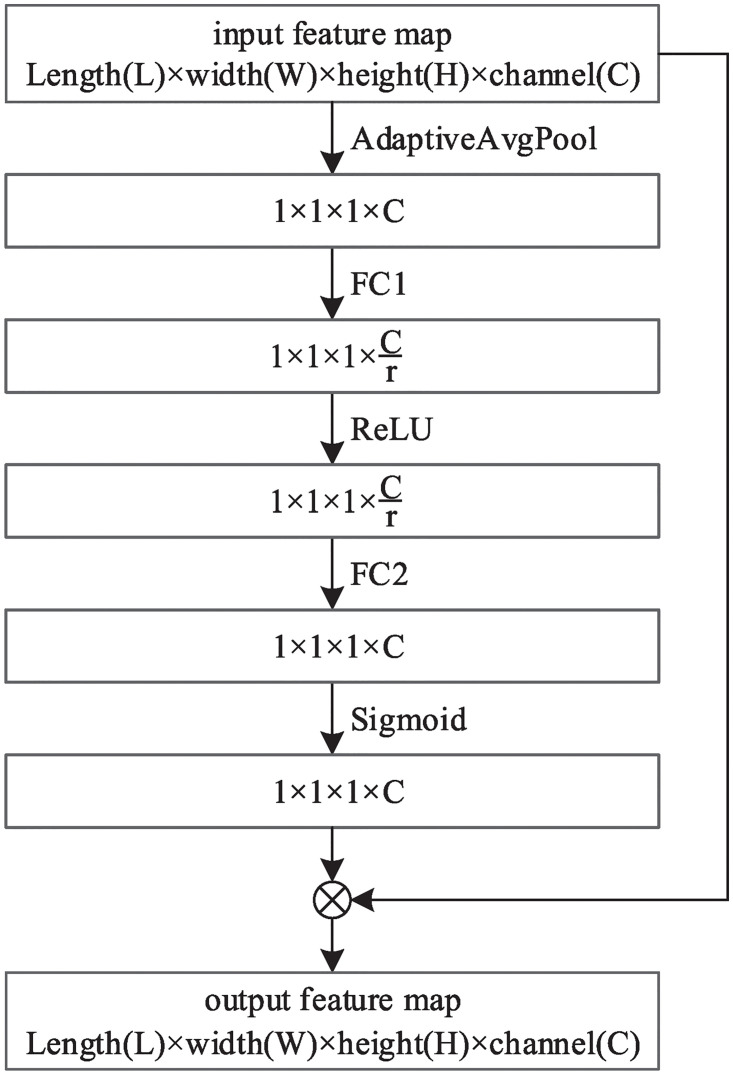
The squeeze-and-excitation unit.

The Bottle2SEneck is the basic module of Res2SENet. Bottle2SEneck involves residual-like connections and a squeeze-and-excitation unit within a single residual block and represents multi-scale features at a granular level. The Bottle2SEneck module is composed of a Res2Net block and a squeeze-and-excitation unit, the structure is shown in Fig 2, where the *x*_*i*_, *y*_*i*_(*i* = 1, 2, 3, 4) represent split feature map, and the 3 × 3 × 3 represents convolution layer with a convolution kernel size of 3 × 3 × 3, each convolution layer is followed by a batch normalization layer and a ReLU layer. The structure of the squeeze-and-excitation unit is shown in Fig 3.

Bottle2SEneck first extracts features from input feature map x using a filter of size 3 × 3 × 3, and splits the output feature map into 4 groups on average according to the channel dimension, which is represented as *x*_1_, *x*_2_, *x*_3_, *x*_4_ in Fig 2. It is worth noting that the spatial size of *x*_1_, *x*_2_, *x*_3_, *x*_4_ are the same. Then the feature map subset *x*_*i*_ is added to *y*_*i*−1_, the output of previous filter *K*_*i*−1_, and fed into the filter *K*_*i*_ to obtain the output feature map *y*_*i*_, which can be expressed by formula as:

$$\begin{array}{c} y_{i}=\{\begin{array}{cc} K_{i}x_{i}, & i=1;\\ K_{i}\left(y_{i-1}+x_{i}\right), & 2\leq i\leq 3;\\ x_{i}, & i=4. \end{array} \end{array}$$ (1)

Next, concatenate *y*_*i*_(*i* = 1, 2, 3, 4) in the channel dimension. In the Bottle2SEneck, we omit the convolution for the fourth split, which can reuse the features; we use three small filters (convolution kernel size 3 × 3 × 3, channel number *C*/4) instead of a large filter (convolution kernel size 3 × 3 × 3, channel number *C*) to increase the receptive field of each output feature map, enable the network to fully extract global and local features and have stronger multi-scale representation capability while maintaining a computational load similar to the networks composed of modules with a large filter. The split and concatenate strategy can make convolution more efficient in processing features.

The squeeze-and-excitation unit structure is shown in Fig 3, it consists of two processes: 1) the squeeze process, which integrates global features through adaptive average pooling; 2) the excitation process, which is implemented through fully connected layer FC1-ReLU-fully connected layer FC2-Sigmoid structure, where r is reduction ratio with a value of 16 in this paper. The excitation process can fully capture the interchannel dependence according to the information gathered in the squeeze process, that is, the channel weight which contains abundant nodule information is significant, but the channel weight which does not contain nodule information is small. Finally, the output (the weight of each channel) generated by the excitation process is multiplied with the feature map of the corresponding channel in the initial input to emphasize the characteristics of the pulmonary nodules.

### Nodule candidate detection network

#### Network structure

The proposed network for detecting nodule candidates in low dose CT scanning is shown in Fig 1(a). The network utilizes region proposal network structure [17–19], and according to the characteristics of this detection task, the scales of anchors in the network are set to 5,10,20. Specifically, the network consists of two parts: the backbone part Res2SENet and the detection part. Since Res2SENet is mainly made up of Res2Net modules and the squeeze-and-excitation units(SE), it is named Res2SENet. The input of the network is a cropped CT image cube with dimensions (*length* × *width* × *height* × *the number of channels*) of 128 × 128 × 128 × 1.

The backbone part Res2SENet of the nodule candidate detection network consists of five stages, the first stage includes two convolutional layers, the second to fifth stages include a max-pooling layer and several Bottle2SEneck modules, respectively, the specific number of modules is shown in Fig 1(a). Among them, the max-pooling layer is used to downsample, reducing the size of the feature map, the Bottle2SEneck module is used to change the number of channels without changing the feature map size. Here we use *c*_*i*_ to represent the output feature map of stage *i*.

Feature pyramid network (FPN) [20] structure increases computational cost and results in enormous runtime latency due to many additional convolutions and detection branches involved. Therefore, in the detection part of the network, we introduced two effective modules, that is, the context enhancement module (CEM) and the spatial attention module (SAM). CEM can integrate multi-scale feature information and enhance feature discrimination. In the CEM, we respectively upsample *c*_4_, *c*_5_ through deconvolution, and concatenate the obtained feature maps with *c*_3_ in the channel dimension. Compared with previous FPN structure, our proposed CEM only involves two deconvolution layers and one feature maps concatenation operation, which reduces the computational cost while ensuring the network effect. SAM performs softmax operation on the feature map from upper layer to get the spatial attention map, and multiplies the spatial attention map with feature map from lower layer to make the network pay more attention to the regions of interest.

We added two Bottle2SEneck modules after SAM and set dropout layer [21] to prevent the phenomenon of overfitting. Finally, the output of the dropout layer is taken as the input of the region proposal network. The output of the region proposal network includes the predicted probability *p* for current anchor being a nodule, and the spatial information (coordinate [*x*, *y*, *z*] and diameter *d*) of nodule candidates.

#### Loss function

The binary class label of each anchor box is assigned based on its intersection over union (IOU) with the target nodule. If the IoU is higher than 0.5, the anchor box is labeled as a positive sample; if the IoU is lower than 0.02, the anchor box is labeled as a negative sample; other anchors that are neither positive nor negative will be neglected during training process. Our loss function consists of classification loss and regression loss. For each labeled anchor box, the multitask loss function is defined as follows:

$$\begin{array}{c} L\left(p_{i},t_{i}\right)=\lambda L_{cls}\left(p_{i},p_{i}^{*}\right)+p_{i}^{*}L_{reg}\left(t_{i},t_{i}^{*}\right). \end{array}$$ (2)

We set the weight parameter λ to 0.5, $L_{cls}\left(p_{i},p_{i}^{*}\right)$ is the classification loss calculated by the binary cross-entropy loss function (*CrossEntropy*)_*binary*_, $L_{reg}\left(t_{i},t_{i}^{*}\right)$ is the regression loss calculated by the $smooth_{l_{1}}$ loss function. $L_{cls}\left(p_{i},p_{i}^{*}\right)$ and $L_{reg}\left(t_{i},t_{i}^{*}\right)$ are defined as follows:

$$\begin{array}{c} L_{\text{cls}}\left(p_{i},p_{i}^{*}\right)=p_{i}^{*}log\left(p_{i}\right)+\left(1-p_{i}^{*}\right)log\left(1-p_{i}\right). \end{array}$$ (3)

$$\begin{array}{c} L_{\text{reg}}\left(t_{i},t_{i}^{*}\right)=\{\begin{array}{cc} 0.5{\left(t_{i}-t_{i}^{*}\right)}^{2}, & \text{if}\;|t_{i}-t_{i}^{*}|<1\\ |t_{i}-t_{i}^{*}|-0.5, & \text{otherwise}. \end{array} \end{array}$$ (4)

In the above formulas, *p*_*i*_ and $p_{i}^{*}$ represent the prediction probability and classification label of an anchor box, respectively. If an anchor box is a positive sample, then its classification label $p_{i}^{*}$ is 1, and if an anchor box is a negative sample, then its classification label $p_{i}^{*}$ is 0. It is easy to see that only positive samples labeled as $p_{i}^{*}=1$ are involved in the calculation of regression loss. *t*_*i*_ and $t_{i}^{*}$ represent the predicted relative coordinates and regression label of an anchor box, respectively, they can defined as:

$$\begin{array}{c} t_{i}=\left(\frac{x-x_{\alpha }}{d_{\alpha }},\frac{y-y_{\alpha }}{d_{\alpha }},\frac{z-z_{\alpha }}{d_{\alpha }},log\left(\frac{d}{d_{\alpha }}\right)\right). \end{array}$$ (5)

$$\begin{array}{c} t_{i}^{*}=\left(\frac{x^{*}-x_{\alpha }}{d_{\alpha }},\frac{y^{*}-y_{\alpha }}{d_{\alpha }},\frac{z^{*}-z_{\alpha }}{d_{\alpha }},log\left(\frac{d^{*}}{d_{\alpha }}\right)\right). \end{array}$$ (6)

where (*x*, *y*, *z*, *d*) are the coordinates and the size of the predicted bounding box, (x^*^, y^*^, z^*^, d^*^) are the coordinates and the size of the ground-truth bounding box, (*x*_*α*_, *y*_*α*_, *z*_*α*_, *d*_*α*_) are the coordinates and the size of the anchor bounding box.

### False positive reduction network

A number of false positive nodules are usually produced in the nodule candidate detection stage. To accurately distinguish true nodules from a large number of nodule candidates, we designed a 3D deep convolutional neural network to further classify the nodule candidates produced in the nodule candidate detection stage to reduce false positives. The network structure is shown in Fig 1(b).

The network consists of five stages. We represent the output feature map of stage *i* in *m*_*i*_. The size (*length* × *width* × *height* × *the number of channels*) of *m*_*i*_ is shown below stage *i* in the figure. In this network, we use convolutional layers and Bottle2SEneck modules to change the number of channels, and use max-pooling layers to downsample, reduce the sizes of feature maps, and apply dropout layers to avoid over-fitting phenomenon, and introduce the binary cross-entropy loss function to optimize.

## Experiments and results

### Datasets

LUNA16 dataset is a subset of the publicly available pulmonary nodule dataset LIDC-IDRI [22]. LUNA16 dataset removes CT images with slice thickness greater than 2.5 mm from LIDC-IDRI dataset, leaving 888 CT images with slice thickness between 0.6 mm and 2.5 mm, spatial resolution between 0.46 mm and 0.98 mm and mean diameter of 8.3 mm. The criterion for determining nodules in the LUNA16 dataset is that at least three out of four radiologists believe that the nodule diameter is greater than 3 mm. Therefore, a total of 1186 positive nodules are annotated in the dataset.

### Preprocessing

For the input CT image, we adopt four automatic preprocessing steps:

1. normalize the voxel value range of pulmonary nodules from the original [-1200, 600] to [0, 1], which is convenient for the neural network to extract effective image features, which is expressed as:
  $$\begin{array}{c} \bar{\text{val}}=\{\begin{array}{cc} 0, & \text{val}<-1200\\ \frac{val-(-1200)}{600-(-1200)}, & -1200\leq \text{val}\leq 600\\ 1, & \text{val}>600. \end{array} \end{array}$$ (7)
  where *val* represents the CT value before normalization and $\bar{val}$ represents the CT value after normalization.
2. remove background based on the CT pulmonary segmentation images provided by the dataset;
3. resample CT images to an isotropic resolution of 1 ×1 × 1 mm;
4. crop the regions of interest for pulmonary nodules.

**Fig 4:**
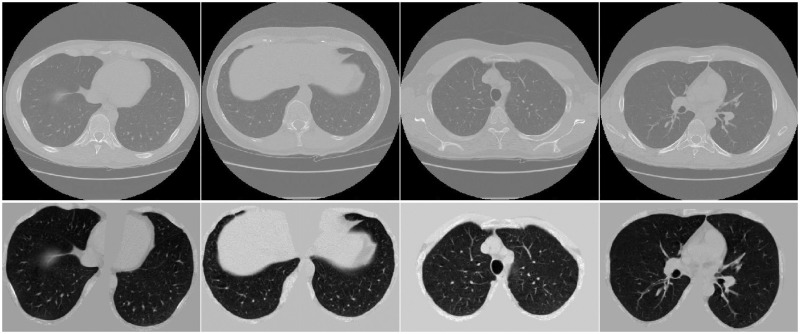
CT images before and after preprocessing. The CT images in Fig 4 were accessed through the links available on this page: https://luna16.grand-challenge.org/Download/ and had not been previously published and/or copyrighted.

First and second lines in Fig 4 show CT images before and after preprocessing, respectively.

### Experimental setup

We experimented with eight Intel(R) Xeon(R) Sliver 4210 CPUs with the master frequency of 2.20G Hz and the memory of 64 G. In this paper, all network models are built by Python 2.7 and accelerated on 2 NVIDIA GeForce RTX 2080Ti graphics cards by Pytorch parallel computing framework. The networks are all optimized by Stochastic gradient descent (SGD) method, where the initial learning rate is set to 0.01, the momentum parameter is set to 0.9, and the weight decay is set to 0.0001. We performed 10-fold cross validation on the dataset.

In the training stage of the nodule candidate detection network, we perform data enhancement after the preprocessing steps by randomly rotating, flipping and cropping, where the cropping scale between 0.75 and 1.25. Set the batch size to 8 and the total number of epochs to 150. After epoch 50, the learning rate is reduced to 0.001, and after epoch 100, the learning rate is reduced to 0.0001. In the testing stage of the nodule candidate detection network, we split the preprocessed CT images into small patches of size 208 × 208 × 208 as inputs to the network, overlapping 32 pixels between neighbouring small patches, that is to say, the distance between the central coordinates of neighbouring small patches is 176. For each CT image, we summarize the nodule candidates obtained by all the small patches, and merge highly overlapping candidates by non-maximum suppression (NMS) [23] with IOU threshold of 0.1 to obtain the detection result.

In the training stage of the false positive reduction network, because the average number of false positive nodules per scan of the nodule candidate detection network is 22, in order to balance the number of positive and negative samples, we amplified the positive samples by 22 times, the amplification methods are consistent with the data enhancement methods in the nodule candidate detection network. We set the batch size to 8 and the total number of epochs to 40. After epoch 10, the learning rate is reduced to 0.001, and after epoch 20, the learning rate is reduced to 0.0001. In the testing stage of the false positive reduction network, the predicted probability obtained by the false positive reduction network is weighted average with the predicted probability obtained by the nodule candidate detection network to get the final classification result, and the calculation formula is as follows:

$$\begin{array}{c} p=\sum_{i=1}^{2}\omega_{i}p_{i} \end{array}$$ (8)

Among them, *ω*_*i*_ is the weight of the prediction probability *p*_*i*_ of the network, we set the weight of the prediction probability of the nodule candidate detection network to 0.2, and the weight of the prediction probability of the false positive reduction network to 0.8.

### Evaluation metrics

Here, the average sensitivity of FROC curve under 7 different false positives (0.125, 0.25, 0.5, 1, 2, 4, 8) is taken as the evaluation result of algorithm performance. The sensitivity formula is as follows:

$$\begin{array}{c} \text{sensitivity}=\frac{TP}{TP+FN} \end{array}$$ (9)

Among them, the TP represents the number of true positive nodules, and the criterion for determining the predicted nodule as true positive nodule is that the center coordinates of predicted nodule are within the ground truth positive nodule. The FN represents the number of false negative nodules, which are the ground truth positive nodules that are not detected.

### Results

**Fig 5:**
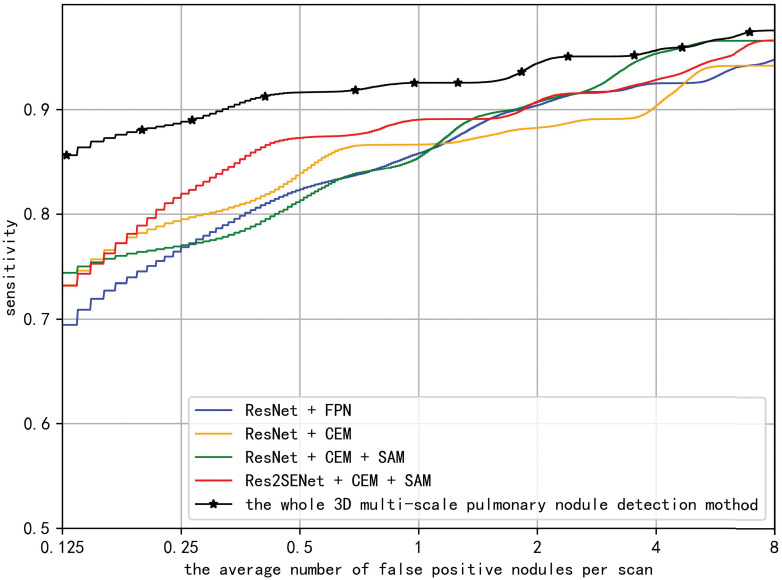
FROC curves.

The performance of the nodule candidate detection network and the whole 3D multi-scale pulmonary nodule detection network are evaluated by FROC curves, average sensitivity, highest sensitivity, and average number of false positives per scan. FROC curves of networks are shown in Fig 5, where the curves are obtained by interpolating true prediction. In Table 1, we compared different 3D nodule candidate detection networks by average sensitivity, highest sensitivity, and average number of false positives per scan. In Table 2, we compared the whole pulmonary nodule detection network with the experimental results of others in terms of average sensitivity.

**Table 1:** Performance comparison of different nodule candidate detection networks.

networks	Average sensitivity	Highest sensitivity	FPs per scan	run time cost per epoch (minutes)
*ResNet* [24] + *FPN* [20]	0.846	0.975	18	31
*ResNet* [24] + *CEM*	0.849	0.958	31	28
*ResNet* [24] + CEM + *SAM*	0.856	0.983	26	29
*Res*2*SENet*+ *CEM*+ *SAM*(*ours*)	0.872	0.983	22	34

**Table 2:** Performance comparison of different methods for pulmonary nodules detection.

methods	Average sensitivity
*Khosravan et al*. [9]	0.897
*Xie et al*. [10]	0.790
*Wang et al*. [12]	0.903
*Ding et al*. [25]	0.891
*Pezeshk et al*. [26]	0.832±0.011
*Li et al*. [27]	0.912
*Proposed*	0.923

To demonstrate the effectiveness of CEM, SAM and Res2Net backbone network in our proposed network structure, we conducted ablation validation experiments on the dataset. The experimental results are shown in Table 1.

To demonstrate the effectiveness of CEM, we compared the combination of FPN and Residual Network(ResNet) [24] with the combination of CEM and ResNet, the experimental results show that the combination of CEM and ResNet has lower highest sensitivity but the average sensitivity is higher, which proves that the CEM with simple structure has comparable performance with FPN. To demonstrate the effectiveness of SAM, we added SAM after CEM, the experimental results show that the average sensitivity of the nodule candidate detection network using SAM increases by 0.7%, the highest sensitivity increases by 2.5%, and the average number of false positives per scan decreases by about 5, indicating that SAM could effectively improve the performance of nodule detection. To demonstrate the effectiveness of Res2SENet backbone network, we combined Res2SENet alternative ResNet, with CEM and SAM. The experimental results show that the average sensitivity of nodule candidate detection network using Res2SENet backbone network increases by 1.6% compared with nodule candidate detection network using ResNet backbone network, and the average number of false positives per scan decreases by about 4, which prove the effectiveness of Res2SENet backbone network.

To demonstrate the effectiveness of the whole 3D pulmonary nodule detection network proposed in this paper, we compared our method with existing state-of-art methods in terms of average sensitivity. The results are shown in Table 2.

**Fig 6:**
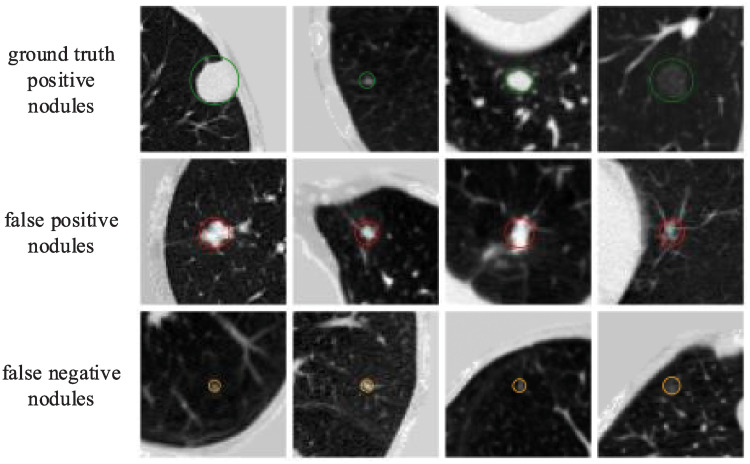
Experimental results. The CT images in Fig 6 were accessed through the links available on this page: https://luna16.grand-challenge.org/Download/ and had not been previously published and/or copyrighted.

For the dataset, the average sensitivity of the proposed method is 0.923, which is higher than the existing state-of-art methods, which shows the superiority of our proposed method. Fig 6 shows the detection results, but because of the 3D nature of CT images, we can only display the slice where the detection center is located. In attention, because pulmonary nodules are relatively small in the slices, we only crop the square area with the detection center as the center and the side length of 64 to visualize. Among them, the first line shows the detected true positive nodules, circled with green circles, and the second line shows the detected false positive nodules, which have very similar characteristics to true positive nodules, circled with red circles, and the third line shows the undetected ground truth positive nodules, i.e., false negative nodules. It is not difficult to see that most of them are extremely small in size, and it is possible to improve the detection performance by special data enhancement on these extremely small nodules. Compared with the traditional methods, our proposed method is not only good for the detection of solid nodules, but also good for the detection of ground glass opacity nodules.

## Conclusion

This paper proposed a 3D multi-scale pulmonary nodule detection method based on deep convolutional neural network. This method consists of two stages: nodule candidate detection stage and false positive reduction stage. In order to fully extract multi-scale features of pulmonary nodules, we combine the Res2Net module with the squeeze-and-excitation unit to build nodule candidate detection network and false positive reduction network. In addition, in the nodule candidate detection network, in order to integrate high-level semantic information with low-level position information, we proposed a context enhancement module with simple structure but excellent performance; To make the network pay more attention to regions of interest, we introduced the spatial attention module after the context enhancement module. Compared with the existing state-of-art pulmonary nodule detection methods, our proposed method has higher average sensitivity and less false positive nodules, and has practical value in the field of pulmonary nodule detection in chest CT sequence image.

Because the 3D multi-scale pulmonary nodule detection method proposed in this paper still has a few extremely small nodules missed diagnosis, it needs to be further optimized in the future to improve the detection performance of this system, for example special data enhancement for these extremely small nodules. In addition, the system can only output the location information of nodules, but in the actual lung cancer screening, the growth site, edge morphology and internal structure of nodules are of great significance for clinical diagnosis. In the future, the sizes, types and characteristics of nodules can be analyzed to provide suggestions for subsequent works.
